# Withdrawn as duplicate: Cobalt Mediates Stage-Specific Toxicity of Metal Mixtures in Cardiovascular-Kidney-Metabolic Syndrome

**DOI:** 10.1093/toxsci/kfaf172

**Published:** 2025-12-15

**Authors:** Wei Zhang, GuangYu Jiang, Ziyan Liu, LianRui Duan, JiaYi Liang, Ziyan Wang, Huiwen Kang, Danyang Huang, Ai Gao

**Affiliations:** Department of Occupational Health and Environmental Health, School of Public Health, Capital Medical University, Beijing 100069, China; Beijing Key Laboratory of environment and aging, Capital Medical University, Beijing 100069, China; Department of Occupational Health and Environmental Health, School of Public Health, Capital Medical University, Beijing 100069, China; Department of Occupational Health and Environmental Health, School of Public Health, Capital Medical University, Beijing 100069, China; Department of Occupational Health and Environmental Health, School of Public Health, Capital Medical University, Beijing 100069, China; Department of Occupational Health and Environmental Health, School of Public Health, Capital Medical University, Beijing 100069, China; Department of Occupational Health and Environmental Health, School of Public Health, Capital Medical University, Beijing 100069, China; Department of Occupational Health and Environmental Health, School of Public Health, Capital Medical University, Beijing 100069, China; Department of Occupational Health and Environmental Health, School of Public Health, Capital Medical University, Beijing 100069, China; Department of Occupational Health and Environmental Health, School of Public Health, Capital Medical University, Beijing 100069, China; Beijing Key Laboratory of environment and aging, Capital Medical University, Beijing 100069, China

This article has been withdrawn due to an error that caused the article to be duplicated. The definitive version of this article is published under https://doi.org/10.1093/toxsci/kfaf168.

